# In Vivo Evidence for Impaired Glymphatic Function in the Visual Pathway of Patients With Normal Pressure Hydrocephalus

**DOI:** 10.1167/iovs.61.13.24

**Published:** 2020-11-17

**Authors:** Henrik Holvin Jacobsen, Tiril Sandell, Øystein Kalsnes Jørstad, Morten C. Moe, Geir Ringstad, Per Kristian Eide

**Affiliations:** 1 Department of Ophthalmology, Oslo University Hospital, Oslo, Norway; 2 Institute of Clinical Medicine, Faculty of Medicine, University of Oslo, Oslo, Norway; 3 Division of Radiology and Nuclear Medicine, Department of Radiology, Oslo University Hospital–Rikshospitalet, Oslo, Norway; 4 Department of Neurosurgery, Oslo University Hospital–Rikshospitalet, Oslo, Norway; 5 Department of Ophthalmology, Vestre Viken Hospital, Drammen, Norway

**Keywords:** cerebrospinal fluid, glymphatic system, visual pathways, normal pressure hydrocephalus, ocular system

## Abstract

**Purpose:**

Impaired ability to remove toxic metabolites from central nervous system may be an important link between cerebral and ophthalmic degenerative diseases. The aim of the present study was to compare the glymphatic function in the visual pathway in patients with idiopathic normal pressure hydrocephalus (iNPH), a neurodegenerative dementia subtype, with a reference group.

**Methods:**

We compared 31 subjects with Definite iNPH (i.e., shunt-responsive) with 13 references in a prospective and observational study. After intrathecal injection of the magnetic contrast agent gadobutrol (Gadovist, 0.5 mL, 1.0 mmol/mL, Bayer Pharma AG), serving as a tracer, consecutive magnetic resonance imaging (MRI) scans were obtained (next 24–48 hours). The normalized MRI T1 signal recorded in the cerebrospinal fluid (CSF) and along the visual pathway served as a semi-quantitative measure of tracer enrichment. Gadobutrol does not penetrate the blood-brain barrier and is thus confined to the extravascular space. Overnight measurements of pulsatile intracranial pressure were used as a surrogate marker for the intracranial compliance.

**Results:**

The tracer enriched the prechiasmatic cistern similarly in both groups, but clearance was delayed in the iNPH group. Moreover, both delayed enrichment and clearance of the tracer were observed in the visual pathway in the iNPH subjects. The enrichment in the visual pathway and the CSF correlated. Individuals with elevated pulsatile intracranial pressure showed reduced enrichment within the visual pathway.

**Conclusions:**

There was delayed enrichment and clearance of a tracer in the visual pathway of iNPH patients, which suggests impaired glymphatic function in the visual pathway in this disease.

The concept of a glymphatic system in the brain was first introduced in 2012.^1^ It refers to a paravascular, pseudolymphatic clearance route for solutes, which plays an important role in removing neurotoxic molecules such as amyloid-β and tau from the central nervous system (CNS).^1^^–^^3^ In 2015, it was hypothesized that the glymphatic system extended to the visual pathway.^4^^,^^5^ Several studies have since supported this hypothesis.^6^^–^^10^ In 2019, we published novel in vivo findings indicating the existence of a glymphatic system in the human visual pathway.^11^ Using a magnetic resonance imaging (MRI) contrast agent (gadobutrol) as a tracer molecule, we demonstrated enrichment within the visual pathway. Because this tracer is confined to the extravascular space by the blood-brain barrier, it can serve as a marker of molecular motions along the glymphatic pathway in humans.

A pathophysiological link between the neurodegenerative nature of dementia and degenerative ophthalmic disorders, such as age-related macular degeneration and glaucoma, has been proposed.^12^^,^^13^ Neuronal damage from neurotoxins, such as amyloid-β, may be a common pathogenic factor.^14^^,^^15^ The glymphatic system could be an important element in this regard. Indeed, impairment of the glymphatic system in the retina and optic nerve of rodents has been demonstrated in glaucoma.^7^^,^^8^

An increased prevalence of glaucoma has been found in idiopathic normal pressure hydrocephalus (iNPH), a form of neurodegenerative disease and dementia subtype^14^^,^^16^^–^^19^ histologically overlapping with Alzheimer's disease.^20^ Moreover, contrast-enhanced MRI of iNPH has indicated impaired glymphatic function in the brain.^21^^,^^22^ Still, to the best of our knowledge, the glymphatic system of the visual pathway has not been studied in iNPH. The purpose of this study was to assess the glymphatic function in the visual pathway of iNPH patients by means of MRI and an intrathecally administered cerebrospinal fluid (CSF) tracer.

## Method

### Approvals

The study was approved by the Institutional Review Board of Oslo University Hospital (reference 2015/1868) and the South-Eastern Norway Regional Committee for Medical and Health Research Ethics (reference 2015/96). The study was conducted in compliance with the tenets of the Declaration of Helsinki, and all participants provided written informed consent. Because gadolinium-based MRI contrast agents are not approved for intrathecal use, approval was also obtained from the Norwegian National Medicine Agency (reference 15/04932-7).

### Patients

In this prospective and observational study, we consecutively included patients from a cohort of individuals undergoing intrathecal contrast-enhanced MRI, as part of a workup for tentative CSF disorders within the Department of Neurosurgery, Oslo University Hospital–Rikshospitalet, Norway. The MRI scans were acquired during the period from October 2015 to January 2018. From the original cohort, two smaller cohorts were selected and compared: (1) Individuals with Definite iNPH, that is, patients with Probable iNPH according to European-American guidelines^23^ and Definite iNPH according to Japanese guidelines.^24^ Individuals with Definite iNPH show a clinical improvement after shunt surgery. (2) The reference (REF) subjects had undergone clinical workup of suspicious symptoms but without evidence of CSF disturbance, pathological overnight intracranial pressure (ICP) measurements, or indication for surgical treatment. They were therefore regarded as close to healthy in the present study context and could serve as a reference group. The REF individuals who were closest in age with the iNPH subjects were included.

### MRI Protocol

Three-dimensional, T1-weighted volume scans were acquired with a 3 Tesla Philips Ingenia MRI scanner (Philips Medical Systems, Best, The Netherlands) with identical imaging protocol settings at all time points. We reconstructed 184 overlapping slices to 368, each 1 mm thick. The method is described in detail in a previous article.^22^ A precontrast MRI scan was followed by consecutive MRI scans, which were acquired after intrathecal injection of gadobutrol. The participants remained in a supine position until the last scan of the first day at about 4 p.m. (they could then move freely). Scans were obtained at the following postinjection time points: 30 minutes, one hour, two hours, four hours, six hours, 24 hours, and 48 hours. All participants underwent MRI scanning up to 24 hours; additionally, nine of 31 iNPH patients and 12 of 13 reference subjects had a scan obtained after 48 hours.

### Image Analyses

**Figure 1.**
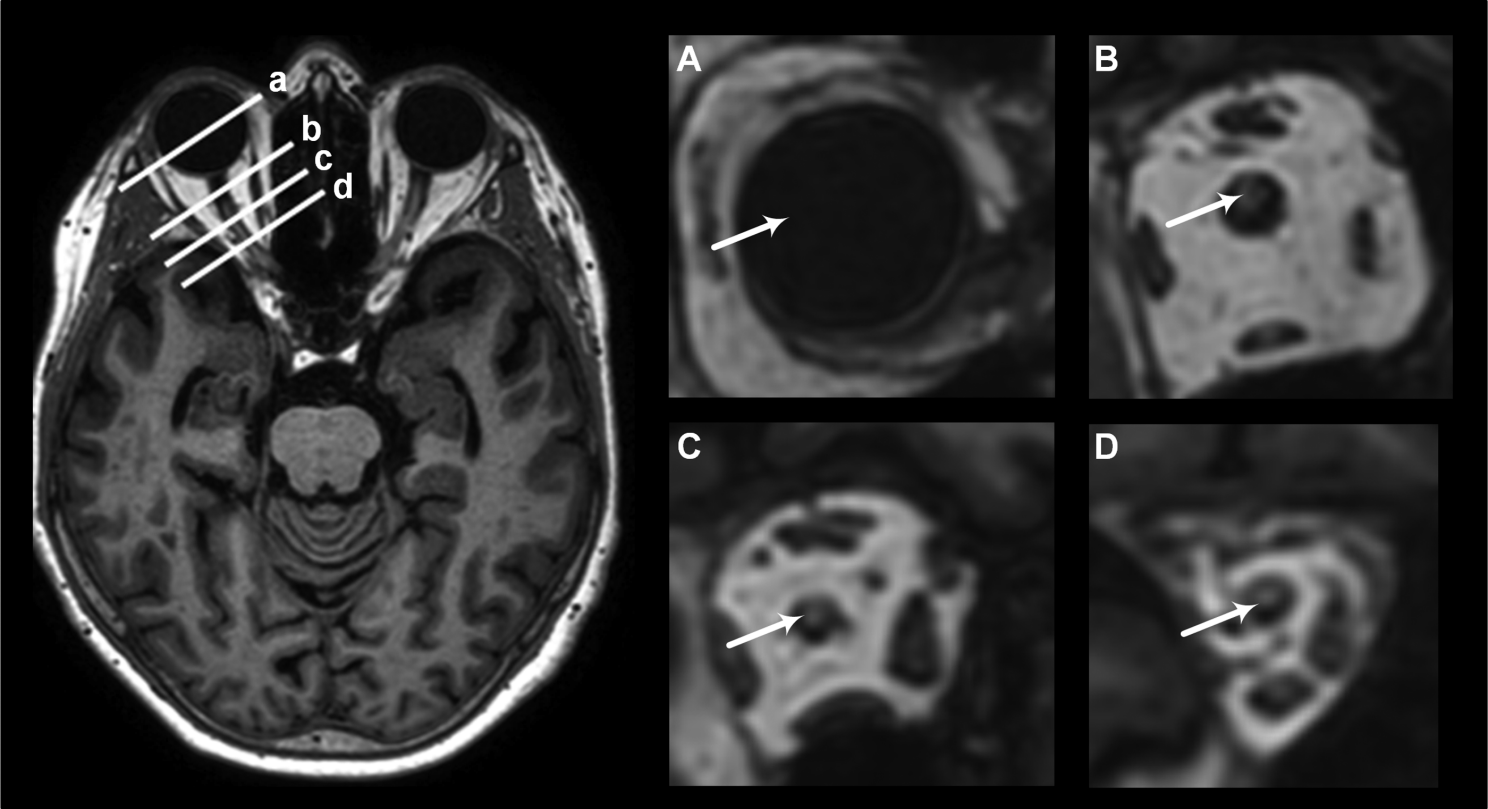
Anatomic location for placement of ROIs in the intraorbital segment of the visual pathway in iNPH using T1-MRI. The figure demonstrates the approximate intraorbital location along the visual pathway used for placement of the ROI. The left image provides an anatomical overview in the axial plane where the letters **a–d** correspond with the magnified coronal slices **A–D**, shown in the images to the right. The following locations are shown and indicated by *arrows*: Vitreous body (**a/A**), retrobulbar part of optic nerve (**b/B**), mid part of optic nerve (**c/C**), and posterior part of optic nerve (**d/D**).

**Figure 2.**
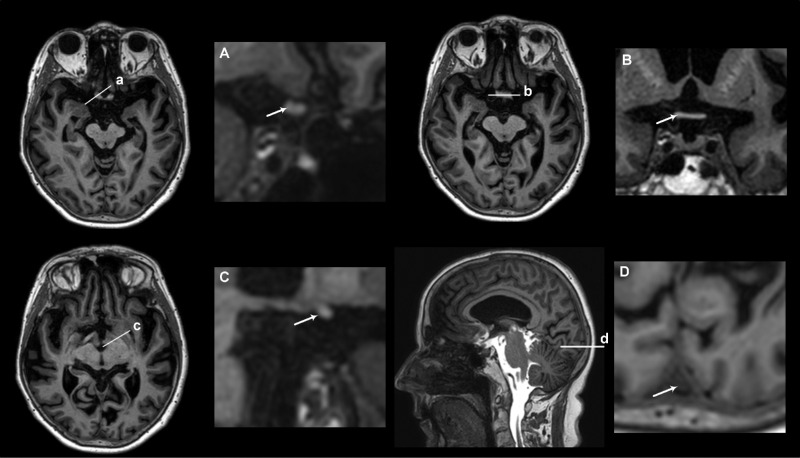
Anatomic location for placement of ROIs in the intracranial segment of the visual pathway in iNPH using T1-MRI. The figure demonstrates the approximate intracranial location along the visual pathway used for placement of the ROI. An anatomic overview is shown in the axial and sagittal planes where the letters **a–d** correspond with the magnified coronal slices (**A–D**). The following locations are shown and indicated by *arrows*: prechiasmatic part of optic nerve (*arrow*) (**a/A**), optic chiasma (**b/B**), optic tract (*arrow*) (**c/C**), and primary visual cortex and superior sagittal sinus (*arrow*) (**d/D**).

For each time point bilateral regions of interest (ROIs) (with the exception of a singular ROI in the midline of optic chiasm) were placed along the visual pathways in the following locations: Within the intraorbital segment, ROIs were placed in the vitreous body and in three separate sections along the intraorbital segment of the optic nerve (retrobulbar, middle, and posterior part), see Figure 1. For the intracranial segment, ROIs were placed in the prechiasmatic segment of the optic nerve, CSF near optic chiasm, optic chiasm, optic tract, and primary visual cortex (Fig. 2). Examples of placements of ROIs are shown in Supplementary Figure S1. Each ROI provides a mean of T1 signal units (SU) at the image grayscale. After normalization to a reference ROI (placed in the superior sagittal sinus), the SU can be compared between time points and study subjects. The normalized T1 SU reflects the enrichment of the tracer and serves as a semiquantitative measure of this. We divide into an enrichment phase and a clearance phase reflecting the increase or decline in tracer enrichment, respectively. The percentage change in tracer enrichment from a precontrast level is compared between the two groups. For simplicity, however, tracer enrichment is referred to as an absolute value instead of percentage change when comparing the two groups.

To better visualize the visual pathway structures, we post-processed the MRI volume scans using the radiology software (Sectra Picture Archiving and Communication System (IDS7; Sectra AB, Linköping, Sweden)) to perform multiplanar reconstructions in the oblique coronal plane perpendicular to the long axis of the optic nerve and optic tract. The ROIs in the optic chiasm and primary visual cortex were placed using standard coronal and axial sections, respectively. Scans of inferior quality, typically due to motion artifacts, were excluded.

### Intrathecal Administration of Gadobutrol

All subjects received an intrathecal injection of 0.5 ml of 1.0 mmol/ml gadobutrol (Gadovist, 1.0 mmol/mL; Bayer Pharma AG, Berlin, Germany). The lumbar puncture was performed by an interventional neuroradiologist under X-ray guidance. A more comprehensive description of the intrathecal administration routine is given in a previous article.^22^

### Overnight Measurements of Mean ICP Wave Amplitude as Indicator of Intracranial Compliance

As part of the clinical routine within the Department of Neurosurgery,^25^ we perform overnight monitoring of the pulsatile ICP, that is, the amplitude of the single ICP waves.^25^ In short, the ICP is measured continuously using a Codman ICP MicroSensor (Codman, Johnson & Johnson, Raynham, Massachusetts) placed 1 to 2 cm into the brain parenchyma, and the cardiac beat-induced single ICP waves are identified. Over consecutives six-second windows, the mean ICP wave amplitude (MWA) is determined. Overnight MWA scores defined as “Above threshold” are 4 mm Hg in average and >5 mm Hg in 10% of recording time.^25^ The MWA is measured as a surrogate marker of intracranial pressure-volume reserve capacity (i.e., intracranial compliance).^26^ This measurement provides information about the capacity of the intracranial compartment to adapt to intracranial volume changes.^27^

### Statistics

Continuous data were described as mean with standard deviation or mean with standard error. Differences between continuous data were assessed with the independent sample *t*-test. To assess impact of age differences between groups, we performed multivariate analysis adjusting for age. From this model, we established trend plots were age of both groups adjusted to 65 years (Supplementary Figs. S2 and S3). Pearson's correlation coefficient test was used to determine correlation. For statistical analyses we used SPSS version 22 (IBM Corporation, Armonk, NY, USA) or Stata/SE version 15.0 (StataCrop LLX, College Station, TX, USA). Statistical significance was accepted at the 0.05 level.

## Results

### Patient Cohorts

We included 31 patients with Definite iNPH and 13 REF subjects. Despite our attempt to match ages, the two groups were significantly different in terms of age (Table 1). There were no adverse effects from gadobutrol or related to the injection procedure. The safety of intrathecal administration of gadobutrol has previously been reported.^28^^,^^29^

**Table 1.** Information About the iNPH Cohort and the References

	iNPH	Reference	*P* Value
Number of patients	31	13	
Sex (F/M)	9/22	9/4	0.01
Age (years)	71.0 ± 6.2	49.6 ± 11.2	<0.001
BMI (kg/m^2^)	26.7 ± 4.0	26.7 ± 4.9	ns
Symptoms reported by patient or family			
Gait disturbance	31 (100%)	0	<0.001
Urinary incontinence	22 (71%)	0	<0.001
Cognitive impairment	28 (90%)	3 (23%)	<0.001
Headache	1 (3%)	11 (85%)	<0.001
Dizziness	6 (19%)	5 (38%)	ns
Lethargy	4 (13%)	5 (38%)	ns
Duration of symptoms (years)	3.1 ± 2.6	3.9 ± 2.8	ns

Data presented as quantities and means (± standard deviation). BMI, body mass index; SD, standard deviation; ns, not significant.

### Changes in Enrichment and Clearance of Tracer in Cerebrospinal Fluid and Visual Pathway

**Figure 3.**
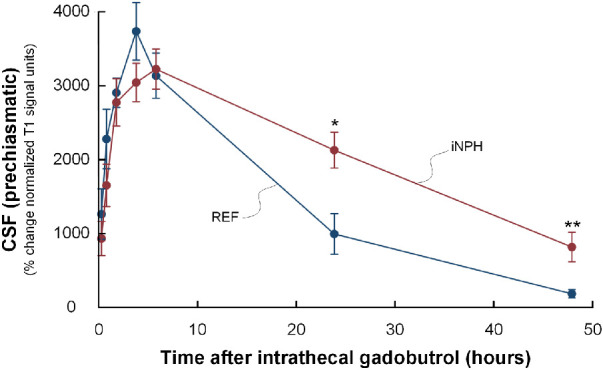
Trend plots of time-dependent enrichment of tracer in cerebrospinal fluid. The figure shows the percentage changes in tracer enrichment (i.e., normalized T1 signal units) within cerebrospinal fluid (CFS) space of prechiasmatic cistern in references (*blue lines*) and iNPH patients (*red lines*). There were no significant difference between the groups during the enrichment phase, whereas during the clearance phase, tracer enrichment were higher after 24 and 48 hours in iNPH, indicative of delayed clearance of tracer from the CSF space in iNPH subjects. *Error bars* refer to standard error (SE). Significant differences between the groups was assessed by the independent *t*-test; *P < 0.05, **P < 0.01.

Both groups displayed an early, marked enrichment of tracer in the prechiasmatic CSF but without differences between the groups. However, the clearance of the tracer within the prechiasmatic CSF space was delayed in iNPH, as shown by higher tracer enrichment remaining in the clearance phase at 24 and 48 hours (Fig. 3).

**Figure 4.**
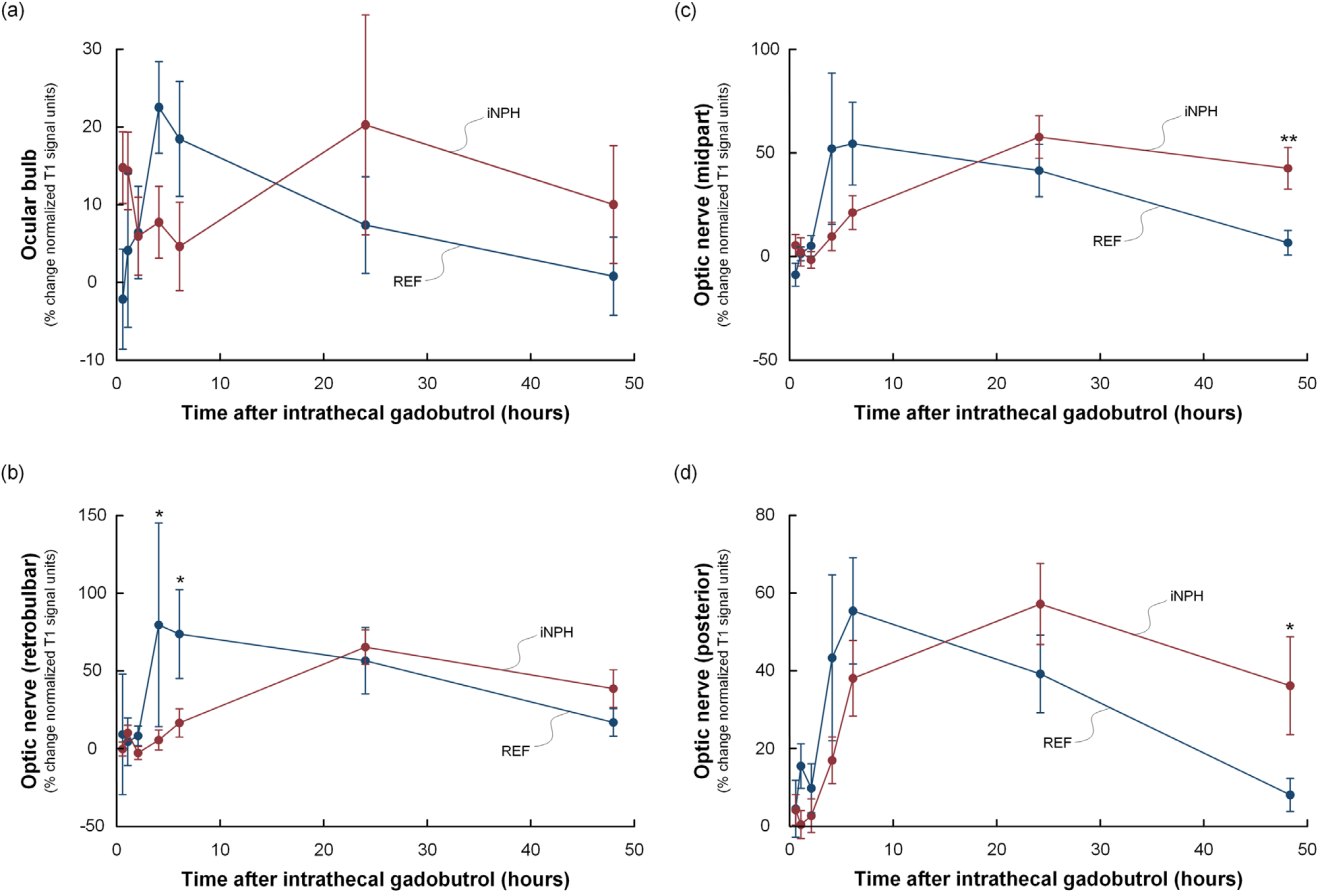
Trend plots of time-dependent tracer enrichment in orbital visual pathway structures. The figure shows the percentage changes in tracer enrichment (i.e., normalized T1 signal units) within the orbital compartment, including (**a**) ocular bulb, (**b**) retrobulbar optic nerve, (**c**) mid part of optic nerve, and (**d**) posterior part of optic nerve in references (*blue line*), and iNPH patients (*red line*). In iNPH cases the tracer enrichment was delayed in the retrobulbar part of the optic nerve shown as significantly lower tracer enrichment at four to six hours (Table 2). A delay in clearance of tracer was seen in the mid and posterior parts, as shown by higher tracer enrichment in the clearance phase at 48 hours (Table 2). *Error bars* refer to 95% CI. Significant differences between the groups at specific time points were assessed by independent *t*-test; *P < 0.05, **P < 0.01.

Within the intraorbital compartment of the optic nerve (Fig. 4, Table 2), we observed delayed enrichment in the retrobulbar part in the iNPH group, as shown by lower tracer enrichment in the enrichment phase at four and six hours. No between-group differences in enrichment of the mid or posterior parts of the optic nerve were observed. The clearance of tracer, however, was delayed in the mid and posterior parts in the iNPH group, as was demonstrated by a higher tracer enrichment remaining in the clearing phase at 48 hours.

**Table 2.** Percentage Change in Tracer Enrichment After Intrathecal Gadobutrol at All Time Points

	30 Minutes	1 Hour	2 Hours	4 Hours	6 Hours	24 Hours	48 Hours
Cerebrospinal fluid (prechiasmatic)							
REF	1260 ± 763	2274 ± 801	2900 ± 661	3730 ± 1029	3129 ± 1099	992 ± 909	184 ± 189
iNPH	930 ± 1271	1648 ± 1483	2769 ± 1786	3038 ± 1423	3219 ± 1489	2125 ± 1348	815 ± 628
						(*P* = 0.01)	(*P* = 0.005)
Intracranial compartment							
Optic nerve (prechiasmatic)							
REF	0 ± 11	7 ± 16	37 ± 19	91 ± 47	88 ± 61	30 ± 41	8 ± 14
iNPH	4 ± 12	11 ± 18	30 ± 36	59 ± 47	67 ± 51	59 ± 87	27 ± 21
							(*P* = 0.022)
Optic chiasm							
REF	-4 ± 17	−1 ± 29	28 ± 24	73 ± 25	60 ± 36	18 ± 17	5 ± 10
iNPH	2 ± 17	9 ± 43	21 ± 33	35 ± 32	47 ± 41	45 ± 89	14 ± 9
				(*P* = 0.006)			
Optic tract							
REF	−4 ± 10	0 ± 13	41 ± 21	84 ± 45	74 ± 46	25 ± 26	7 ± 10
iNPH	3 ± 18	10 ± 22	28 ± 33	41 ± 28	44 ± 35	46 ± 77	18 ± 21
				(*P* = 0.003)	(*P* = 0.025)		
Primary visual cortex							
REF	−7 ± 8	−9 ± 15	−5 ± 17	5 ± 22	19 ± 48	32 ± 34	12 ± 12
iNPH	1 ± 13	0 ± 17	−1 ± 15	0 ± 16	1 ± 20	54 ± 76	43 ± 26
							(*P* = 0.002)
Orbital compartment							
Optic nerve (retrobulbar)							
REF	9 ± 55	5 ± 30	8 ± 18	80 ± 147	74 ± 95	57 ± 74	17 ± 30
iNPH	0 ± 21	10 ± 20	−3 ± 21	6 ± 31	17 ± 47	66 ± 62	39 ± 38
				(*P* = 0.027)	(*P* = 0.018)		
Optic nerve (midpart)							
REF	−9 ± 11	2 ± 7	5 ± 15	52 ± 82	55 ± 69	42 ± 46	7 ± 20
iNPH	6 ± 25	3 ± 28	−1 ± 20	10 ± 33	22 ± 41	58 ± 57	43 ± 32
							(*P* = 0.005)
Optic nerve (posterior)							
REF	4 ± 16	15 ± 12	10 ± 20	43 ± 53	56 ± 47	39 ± 36	8 ± 14
iNPH	4 ± 18	0 ± 15	3 ± 20	17 ± 30	38 ± 49	57 ± 58	36 ± 40
							(*P* = 0.04)
Vitreous body							
REF	−2 ± 14	4 ± 20	6 ± 21	22 ± 15	19 ± 27	7 ± 22	1 ± 17
iNPH	15 ± 26	14 ± 26	6 ± 28	8 ± 25	5 ± 31	20 ± 79	10 ± 24

The table shows the percentage changes of tracer enrichment (i.e. normalized T1 signal units) within the studied locations of both groups. Measurements with statistical significance are marked with a p-value in parenthesis. Delayed tracer enrichment is seen as lower percentage change in normalized T1 signal units from pre-contrast at 4-6 hours compared with the reference group. Delayed clearance is shown as a remaining higher percentage increase in tracer enrichment in the clearance phase at 24 to 48 hours compared with the reference group. Data presented as mean ± standard deviation. Significant differences between groups at specific time points were assessed by independent *t*-test.

*P < 0.05.

**P < 0.01.

**Figure 5.**
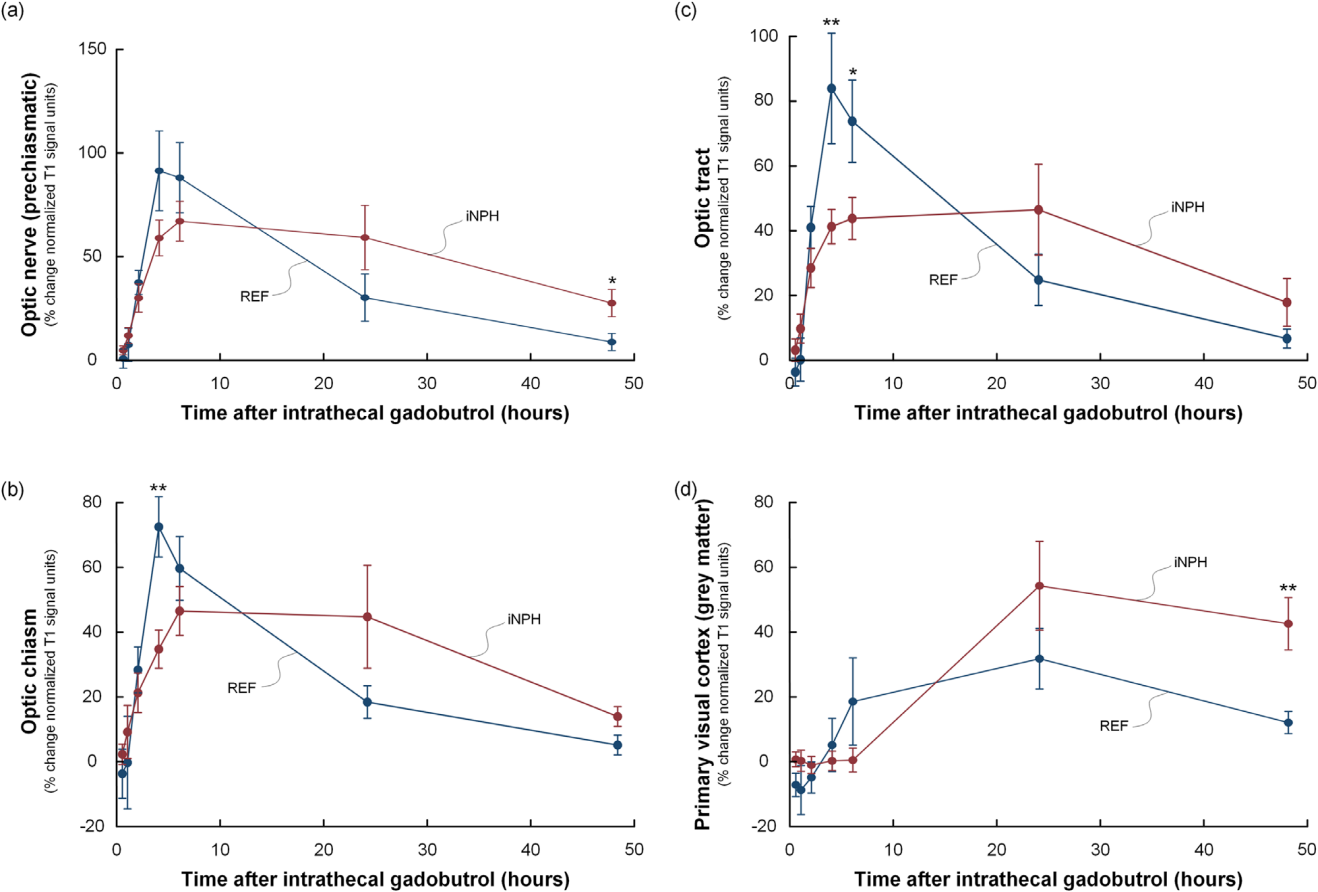
Trend plots of time-dependent tracer enrichment in intracranial visual pathway. The figure shows the percentage changes in tracer enrichment (i.e., normalized T1 signal units) within the intracranial compartment, including (**a**) prechiasmatic optic nerve, (**b**) optic chiasm, (**c**) optic tract, and (**d**) primary visual cortex (gray matter) in references (*blue line*) and iNPH patients (*red line*). In iNPH cases the tracer enrichment was delayed in the optic chiasm and the optic tract, as shown by significantly lower tracer enrichment at four to six hours (Table 2). The clearance of tracer was delayed at 48 hours for the prechiasmatic optic nerve and the primary visual cortex, as shown by more pronounced tracer enrichment in the clearance phase at 48 hours (Table 2). *Error bars* refer to 95% CI. Significant differences between the groups at specific time points were assessed by independent *t*-test; *P < 0.05, **P < 0.01.

Within the intracranial compartment, delayed enrichment was seen in the optic chiasm and optic tract in the iNPH group, as shown by lower tracer enrichment at four hours (optic chiasm) and at four and six hours (optic tract) (Fig. 5, Table 2). The tracer enrichment remained at a higher level in the prechiasmatic optic nerve and the primary visual cortex in the clearing phase at 48 hours of iNPH patients, demonstrating impaired clearance within these regions.

Multivariate analysis adjusting for age gave very similar results and did not change our observations. There was no significant effect of age at any of the locations. Trend plots for percentage change in tracer enrichment of REF and iNPH where both groups were adjusted for age are shown for the intraorbital compartment in Supplementary Figure S2 and for the intracranial segment in Supplementary Figure S3. We observed a strong association between the tracer enrichment within CSF and within the visual pathway (Table 3), which was most apparent at four, six, 24, and 48 hours.

**Table 3.** Correlation Between Change in Enrichment in Cerebrospinal Fluid and Different Regions of the Visual Pathway

	4 Hours	6 Hours	24 Hours	48 Hours
Intracranial compartment				
Optic nerve (prechiasmatic)				
4 hours	*R* = 0.68; *P* < 0.001			
6 hours		*R* = 0.68; *P* < 0.001		
24 hours			*R* = 0.80; *P* < 0.001	
48 hours				*R* = 0.80; *P* < 0.001
Optic chiasm				
4 hours	*R* = 0.50; *P* = 0.002			
6 hours		*R* = 0.52; *P* < 0.001		
24 hours			*R* = 0.70; *P* < 0.001	
48 hours				*R* = 0.57; *P* = 0.008
Optic tract				
4 hours	*R* = 0.52; *P* = 0.001			
6 hours		*R* = 0.60; *P* < 0.001		
24 hours			*R* = 0.75; *P* < 0.001	
48 hours				*R* = 0.81; *P* < 0.001
Primary visual cortex				
4 hours	*R* = 0.32; *P* = 0.05			
6 hours		*R* = 0.47; *P* = 0.002		
24 hours			*R* = 0.80; *P* < 0.001	
48 hours				*R* = 0.79; *P* < 0.001
Orbital compartment				
Optic nerve (retrobulbar)				
4 hours	*R* = 0.48; *P* = 0.01			
6 hours		*R* = 0.42; *P* = 0.01		
24 hours			*R* = 0.72; *P* < 0.001	
48 hours				*R* = 0.54; *P* = 0.011
Optic nerve (midpart)				
4 hours	*R* = 0.45; *P* = 0.017			
6 hours		*R* = 0.49; *P* = 0.002		
24 hours			*R* = 0.71; *P* < 0.001	
48 hours				*R* = 0.76; *P* < 0.001
Optic nerve (posterior)				
4 hours	*R* = 0.60; *P* < 0.001			
6 hours		*R* = 0.46; *P* = 0.004		
24 hours			*R* = 0.77; *P* < 0.001	
48 hours				*R* = 0.79; *P* < 0.001
Vitreous				
4 hours	*R* = 0.22; ns			
6 hours		*R* = 0.31; *P* = 0.048		
24 hours			*R* = 0.67; *P* < 0.001	
48 hours				*R* = 0.40; ns

We observed a significant correlation between percentage change of enrichment (i.e. normalized T1 signal units) in the cerebrospinal fluid and several regions along the visual pathway. This was most evident for the 4, 6, 24, and 48 hours measurements. Ns, not significant; *R*, Pearson correlation coefficient.

### Pulsatile Intracranial Pressure and Enrichment in the Visual Pathway

In 29 iNPH patients and five reference individuals, the mean wave amplitude was measured overnight and categorized as above or below a previously established threshold.^25^ The tracer enrichment at four and six hours was lower for individuals with MWA above threshold (Table 4).

**Table 4.** Differences Between MWA Categories Regarding Tracer Enrichment After Four and Six Hours for Different Regions of Visual Pathway

	Below Threshold (*n* = 5)	Above Threshold (*n* = 29)	*P* **Value**
Cerebrospinal fluid			
4 hours	4401 ± 1307	3019 ± 1444	ns
6 hours	3793 ± 1337	3253 ± 1503	ns
Intracranial compartment			
Optic nerve (prechiasmatic)			
4 hours	132 ± 77	59 ± 47	0.047
6 hours	136 ± 71	69 ± 51	0.016
Optic chiasm			
4 hours	83 ± 27	35 ± 32	0.018
6 hours	82 ± 32	48 ± 41	ns
Optic tract			
4 hours	115 ± 56	41 ± 28	<0.001
6 hours	114 ± 48	45 ± 35	0.001
Primary visual cortex			
4 hours	23 ± 19	−1 ± 15	0.017
6 hours	51 ± 64	0 ± 20	0.001
Orbital compartment			
Optic nerve (retrobulbar)			
4 hours	189 ± 215	6 ± 31	<0.001
6 hours	178 ± 138	17 ± 48	<0.001
Optic nerve (midpart)			
4 hours	124 ± 98	10 ± 33	<0.001
6 hours	115 ± 96	22 ± 42	0.002
Optic nerve (posterior)			
4 hours	73 ± 61	17 ± 30	0.011
6 hours	85 ± 59	39 ± 50	ns
Vitreous			
4 hours	33 ± 12	5 ± 22	0.037
6 hours	45 ± 20	5 ± 31	0.010

The table shows the percentage changes in enrichment (i.e., normalized T1 signal units) at four and six hours for patient categorized with MWA above and below threshold, representing impaired and normal intracranial compliance, respectively. In the group with MWA above threshold, i.e. impaired intracranial compliance and reduced capacity to compensate for an intracranial volume increase, tracer enrichment was reduced within the various locations. No significant difference was seen in the cerebrospinal fluid. Ns, not significant.

## Discussion

The main finding of this study is delayed tracer enrichment and clearance in both orbital and intracranial parts of the visual pathway in individuals with iNPH. Moreover, the tracer distribution within the visual pathway depends on the availability of tracer in the CSF space, and seems to be affected by the intracranial compliance. Age differed between the groups but multivariate analysis adjusted for age gave similar results.

Our group has previously shown that the MRI contrast agent gadobutrol serving as a tracer molecule is well suited for assessing the glymphatic circulation within the brain and visual pathway.^11^^,^^22^^,^^30^ The tracer is a small, hydrophilic molecule with an estimated hydrodynamic diameter of <2.6 nm, which does not cross the blood-brain barrier.^21^^,^^31^ The tracer is therefore confined to the extravascular space. Studies of a glymphatic system in the optic nerve of rodents and humans have shown that tracers injected into the CSF enter the optic nerve via paravascular spaces.^6^^,^^7^^,^^10^ We suggest that the main path from the CSF compartment into the visual pathway for gadobutrol is via paravascular routes, which is a prerequisite for a visual glymphatic system.^1^ Furthermore, the tracer serves as a model for organic molecules with similar properties, and our findings can thus indicate impaired extravascular transport of other substances (e.g., amyloid-β).^32^

### Delayed Paravascular Molecular Transport in iNPH

We interpret the lower tracer enrichment in the visual pathway after four to six hours in iNPH as indicative of delayed paravascular influx, whereas the increased tracer enrichment in the clearance phase after 24 to 48 hours are interpreted as delayed molecular clearance from extravascular spaces. Delayed enrichment and clearance of the tracer suggest that the visual glymphatic circulation is disturbed in iNPH patients.

Lymphatic drainage of small and large molecules appear to be an important outflow route for CSF in rodents.^33^ In a previous human MRI tracer study, drainage to a cervical lymph node was demonstrated after intrathecally injected gadobutrol.^34^ The recently discovered lymphatic vessels in the dura mater^35^^–^^37^ are proposed to be the link between the glymphatic system and the peripheral lymphatic structures.^35^^,^^36^^,^^38^ Both paravascular influx and clearance of macromolecules have been shown to be reduced after blocking these vessels in rodents.^35^^,^^39^ Accordingly, delayed clearance of tracer from the CSF due to reduced lymphatic outflow may be a major determinant for our observations.

The astrocytes and their residing water channels, aquaporin-4 (AQP4), have been shown to be crucial for the exchange of fluids and solutes between the paravascular and interstitial compartments.^1^^,^^40^ Astrogliosis and loss of paravascular AQP4 channels have been found in cortical biopsy specimens of iNPH patients^41^^,^^42^ and may contribute to the delayed tracer transport observed in our study.

### Pulsatile Intracranial Pressure Affects Enrichment

The overnight measurement of pulsatile intracranial pressure, which we refer to as mean ICP wave amplitude, provides a surrogate marker of the intracranial pressure-volume reserve capacity or intracranial compliance.^26^ Impaired intracranial compliance, as indicated by mean wave amplitude above thresholds, has thus been a consistent observation in iNPH individuals who adequately respond to CSF diversion surgery, that is, individuals with definite iNPH.^43^ It can be hypothesized that impaired intracranial compliance and cerebrovascular comorbidity, which also characterizes iNPH,^44^ restrict arterial pulsatility. The latter may in turn impair paravascular molecular movement, resulting in impaired glymphatic function.^45^^,^^46^ In this regard, the present observations of lower tracer enrichment in visual pathway of individuals with pulsatile ICP above threshold are interesting.

### Possible Ophthalmologic Implications

An intriguing hypothesis is the existence of a link between neurodegenerative diseases of the brain and the eye.^12^^,^^13^ Notably, an increased prevalence of glaucoma among iNPH patients has been found.^14^^,^^17^^–^^19^ A pathophysiological link between glymphatic dysfunction and glaucoma has been proposed.^4^ In support of this hypothesis, abnormal glymphatic circulation in glaucoma has been demonstrated in animal models.^7^^,^^8^ Recently, Wang et al.^7^ identified a glymphatic clearance route for amyloid-β in the retina and orbital part of the optic nerve in rodents. In their study, pathological glymphatic flow was observed in glaucomatous mice. In another rodent study, Mathieu et al.^8^ showed that the paravascular influx of a CSF tracer into the optic nerve is reduced in glaucoma, indicating abnormal glymphatic flow. Neurotoxic compounds may accumulate in consequence of impaired glymphatic flow along the visual pathway. An intriguing topic for future studies may be exploring the potential effect of impaired glymphatic circulation on the retinal ganglion cells.

The available literature on a glymphatic system in the visual pathway encompasses the orbital part. Our findings expand on this knowledge and indicate that the glymphatic circulation is also reduced in the intracranial part of the visual pathway.

### Limitations

Limitations of this study were the differences in age and gender between iNPH and references. To our knowledge, there are no studies on the gender in a glymphatic context, and it is difficult to interpret the potential effect on our results. The prevalence of iNPH is strongly associated with age.^47^^,^^48^ Age has been shown to influence the flow of CSF and the glymphatic function.^49^ It is therefore possible that some of the differences between the groups represent age effects. On the other hand, multivariate analysis adjusting for age gave very similar results and did not affect our conclusions. Furthermore, the present results correspond well with previous comparable human tracer-studies of iNPH.^21^^,^^22^ These studies, comparing older iNPH patients to a younger reference group, demonstrated delayed tracer transport in the brain and CSF in the iNPH group. However, differences in additional parameters like speed of gadobutrol propagation, ventricular reflux, and periventricular ependymal enhancement were also observed. These differences are more likely linked to disturbed CSF flow in iNPH. In the current study we use the same MRI tracer and the same MRI protocol, and the study cohorts are similar. On this background, we find it plausible that our findings may, at least partly, be due to delayed molecular transport in iNPH. Even though no indication for surgery was found, the ICP was in normal limits, and MRI showed no major disturbances in the CSF dynamics; a second limitation was that the reference group also was patients, not completely healthy controls. Finally, when positioning of ROIs in small structures like the visual pathway, there is a risk of partial volume averaging effects. To minimize this risk, we postprocessed the MRI scans as described in methods.

## Conclusion

The present observations of delayed enrichment and clearance of a tracer molecule confined to the extravascular space indicate that the glymphatic function is impaired in the visual pathway of iNPH patients. The iNPH patients were older than the controls, but similar results were present after adjusting for age differences. However, future studies should more accurately address the relative effect of age on the decreased glymphatic turnover. Moreover, the study suggests that the enrichment depends on both the tracer availability in the CSF spaces and the intracranial compliance. Reduced glymphatic clearance of a tracer in the visual pathway of iNPH patients suggests that removal of molecules with comparable properties, such as amyloid-β and tau, could also be impaired. This provides a potential pathophysiological explanation for an increased prevalence of glaucoma in iNPH.

## Supplementary Material

Supplement 1
