# Supplementary material for: In Vivo Evidence for Impaired Glymphatic Function in the Visual Pathway of Patients With Normal Pressure Hydrocephalus
Source: Invest Ophthalmol Vis Sci. 2020 Nov 17;61(13):24. doi: 10.1167/iovs.61.13.24 (PMC7683855; doi:10.1167/iovs.61.13.24)
Supplement: Supplement 1 [file iovs-61-13-24_s001.pdf]

Supplementary Material

In vivo evidence for impaired glymphatic function in the visual pathway of patients with normal pressure hydrocephalus

Henrik Holvin Jacobsen, MD[1,2], Tiril Sandell, MD, PhD[1,5] , Øystein Kalsnes Jørstad, MD, PhD[1], Morten C. Moe, MD, PhD [1,2], Geir Ringstad, MD, PhD[3] , Per Kristian Eide, MD, PhD[2,4]

[1]Department of Ophthalmology, Oslo University Hospital, Oslo, Norway
[2]Institute of Clinical Medicine, Faculty of Medicine, University of Oslo, Oslo, Norway
[3]Division of Radiology and Nuclear Medicine, Department of Radiology, Oslo University Hospital - Rikshospitalet, Oslo, Norway
[4]Department of Neurosurgery, Oslo University Hospital — Rikshospitalet, Oslo, Norway
[5]Department of Ophthalmology, Vestre Viken Hospital, Drammen, Norway

Corresponding author: Per Kristian Eide, MD, PhD, p.k.eide@medisin.uio.no

Suppl. Fig. 1. T1-weighed magnetic resonance imaging showing placement of region of interest (ROI)

The images in Suppl Fig. 1 demonstrate the approximate placement of the ROIs in a reference patient. The same placement was used in both groups. Bilateral (except for singular in the optic chiasm) ROIs were placed along both visual pathways in the coronal plane (A-G) and in the axial plane for the primary visual cortex (H). A ROI placed in the axial plane of the superior sagittal sinus (H) was utilized as reference. The same locations were used in each time point. We placed the ROIs in the center of the structures to avoid partial volume averaging defects.

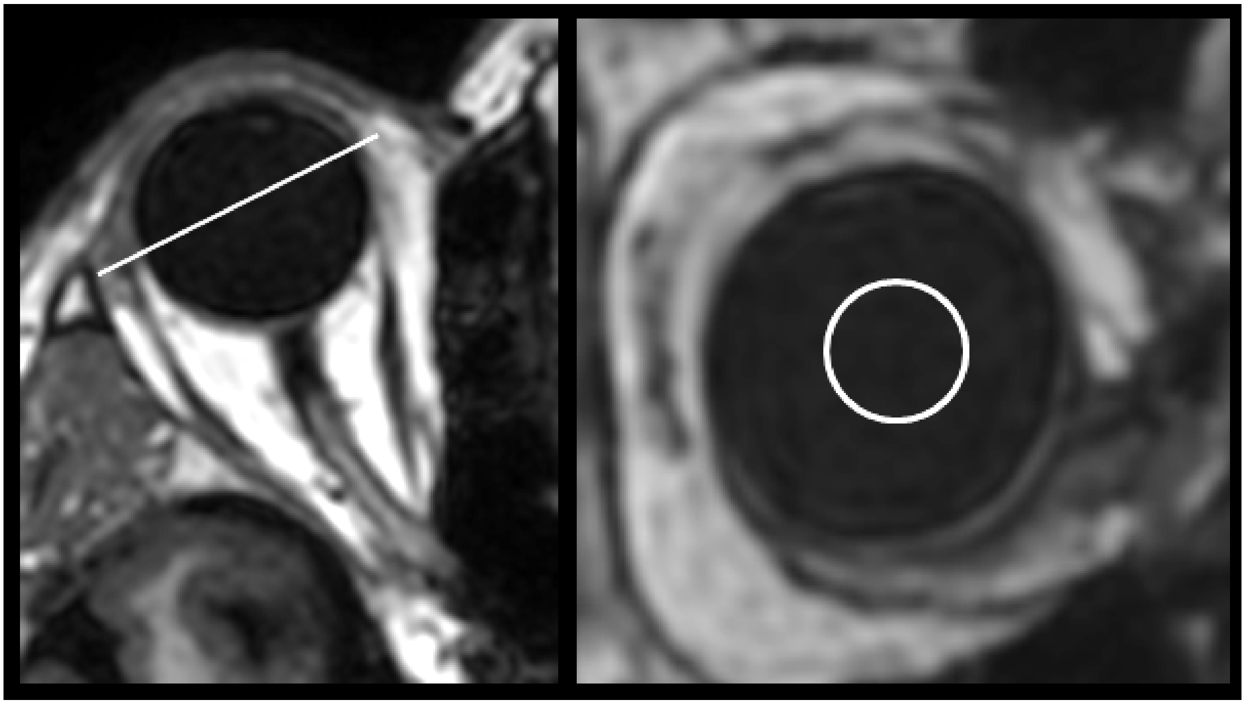

Suppl. Fig. 1A. Placement of region of interest in the vitreous body shown to the right, anatomical overview is shown in the axial slice to the left.

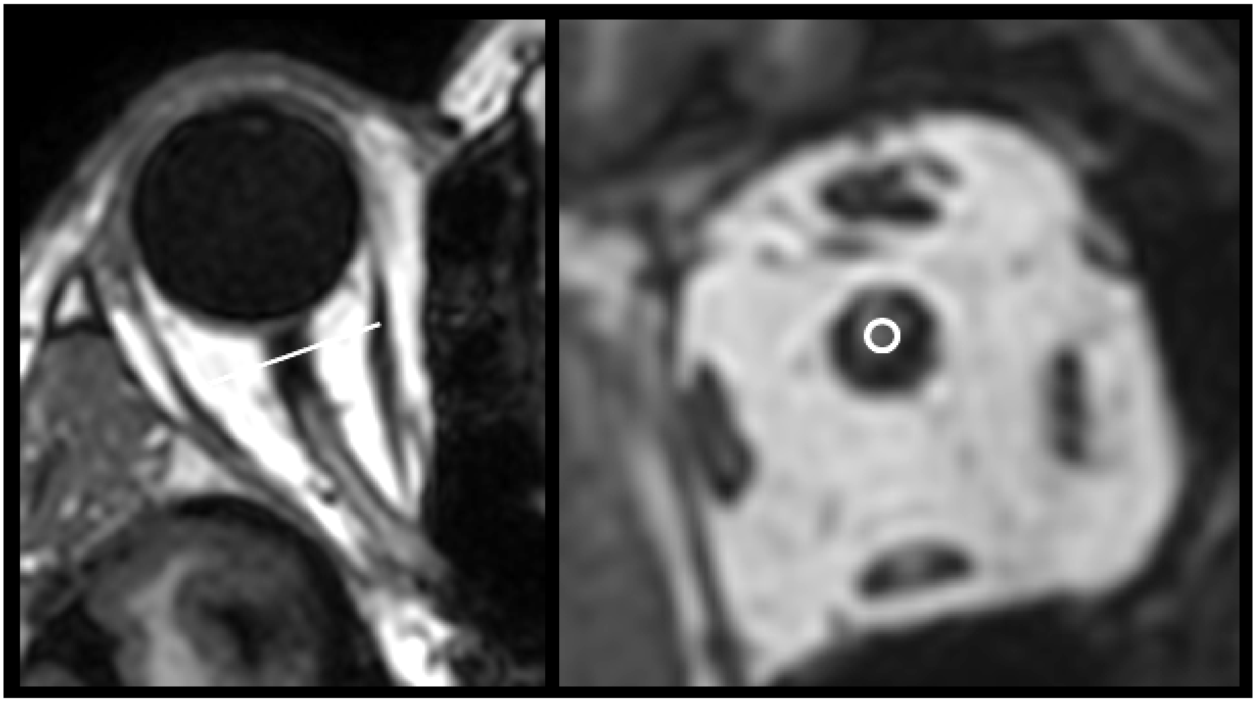

Suppl. Fig. 1B. Placement of region of interest in retrobular part of the optic nerve shown to the right, anatomical overview is shown in the axial slice to the left.

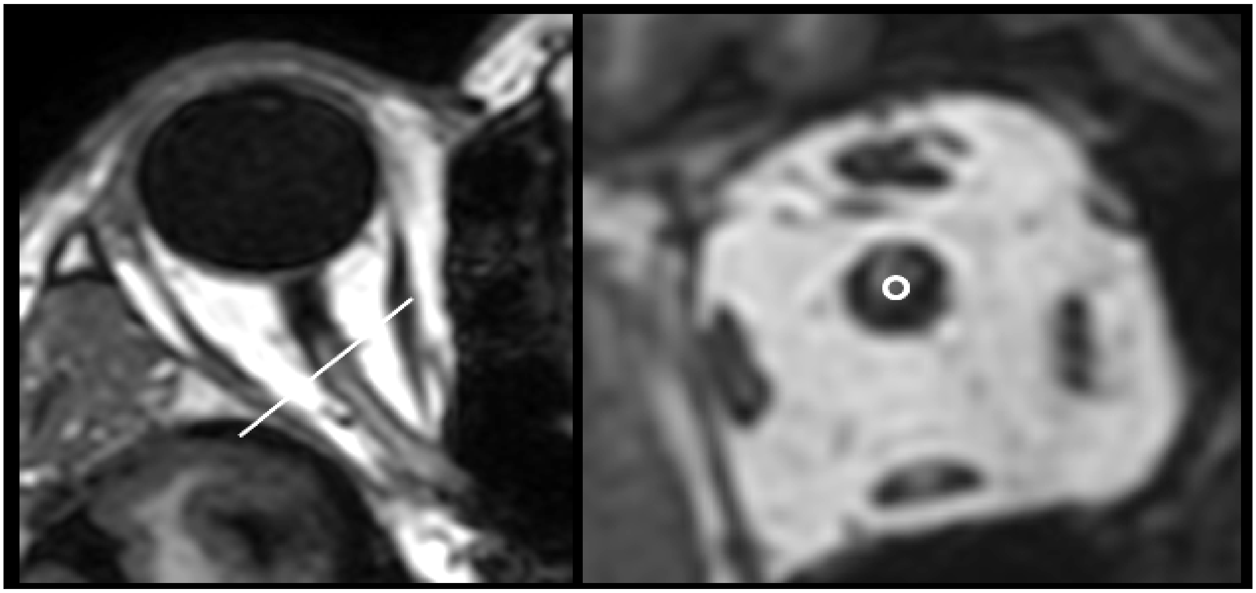

Suppl. Fig. 1C. Placement of region of interest in the mid part of the optic nerve shown to the right, anatomical overview is shown in the axial slice to the left.

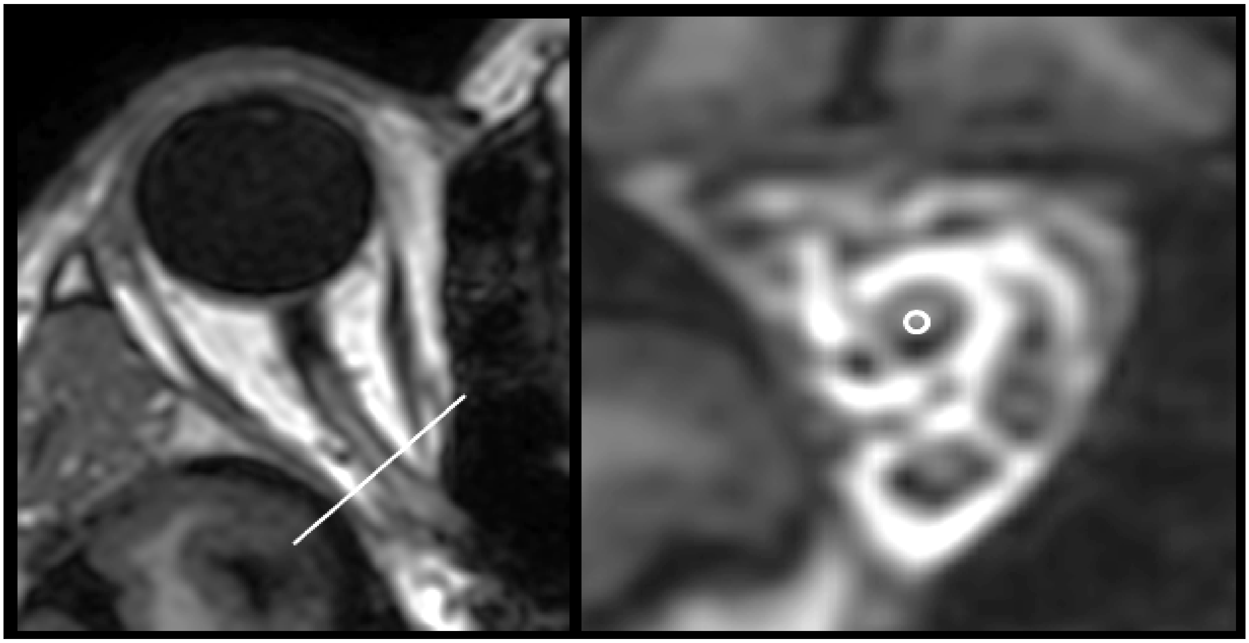

Suppl. Fig. 1D. Placement of region of interest in the posterior part of the optic nerve shown to the right, anatomical overview is shown in the axial slice to the left.

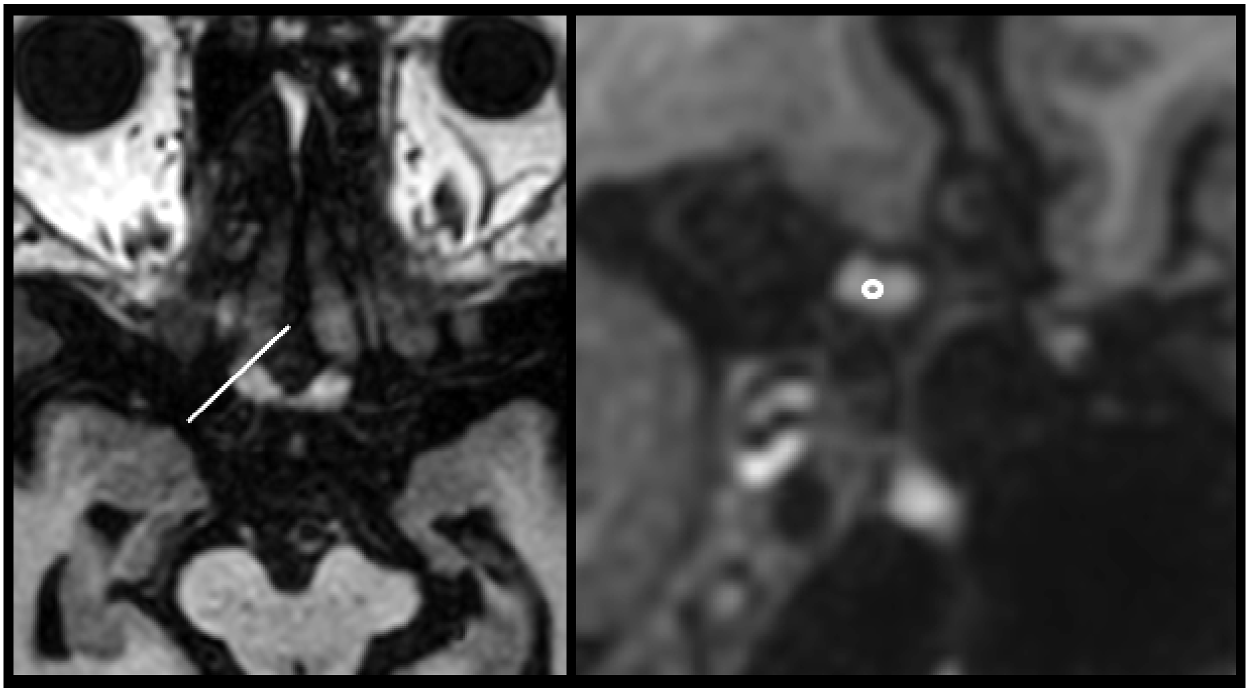

Suppl. Fig. 1E. Placement of region of interest in the prechiasmatic part of the optic nerve shown to the right, anatomical overview is shown in the axial slice to the left.

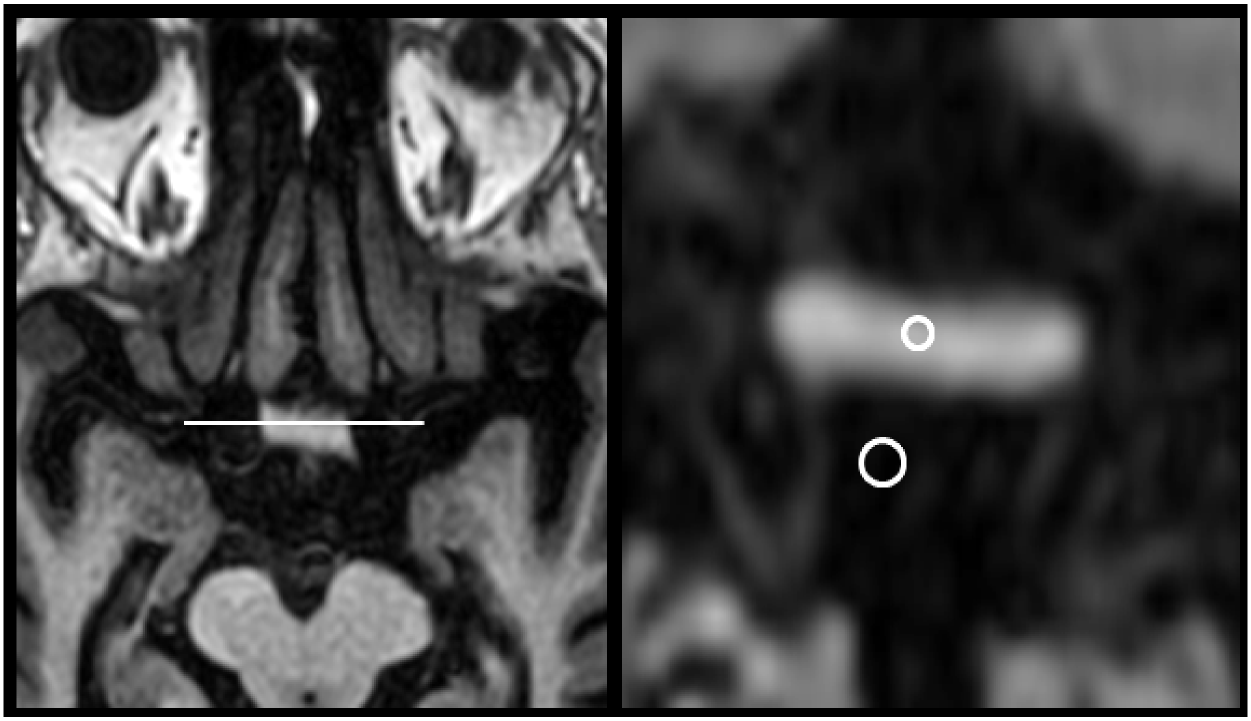

Suppl. Fig. 1F. Placement of region of interest in the optic chiasm and cerebrospinal fluid in the prechiasmatic cistern shown to the right, anatomical overview is shown in the axial slice to the left.

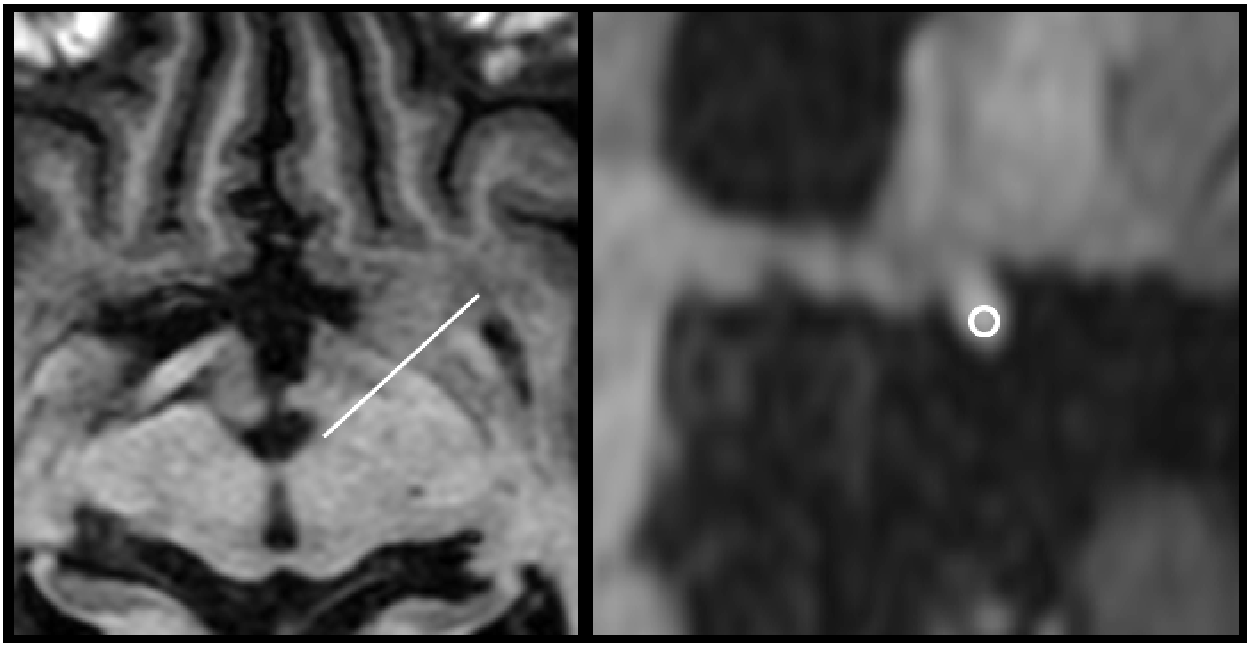

Suppl. Fig. 1G. Placement of region of interest in the optic tract shown to the right, anatomical overview is shown in the axial slice to the left.

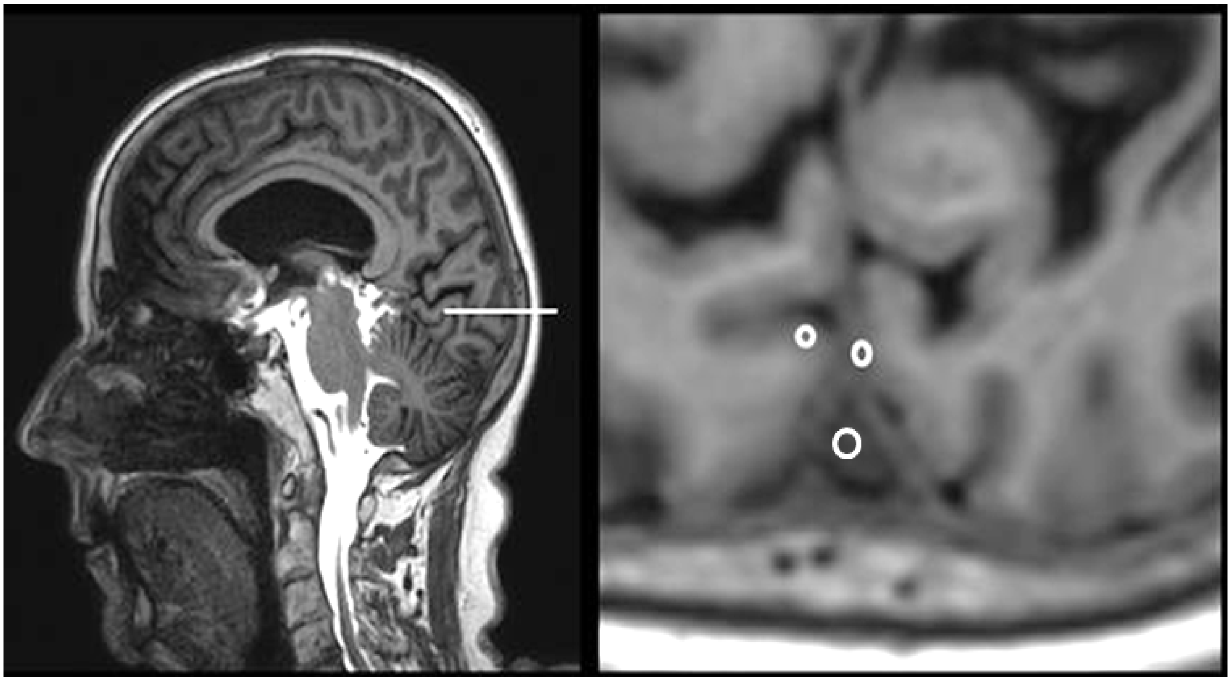

Suppl. Fig. 1H. Placement of region of interest in the primary visual cortex and the superior sagittal sinus shown to the right, anatomical overview is shown in the sagittal slice to the left.

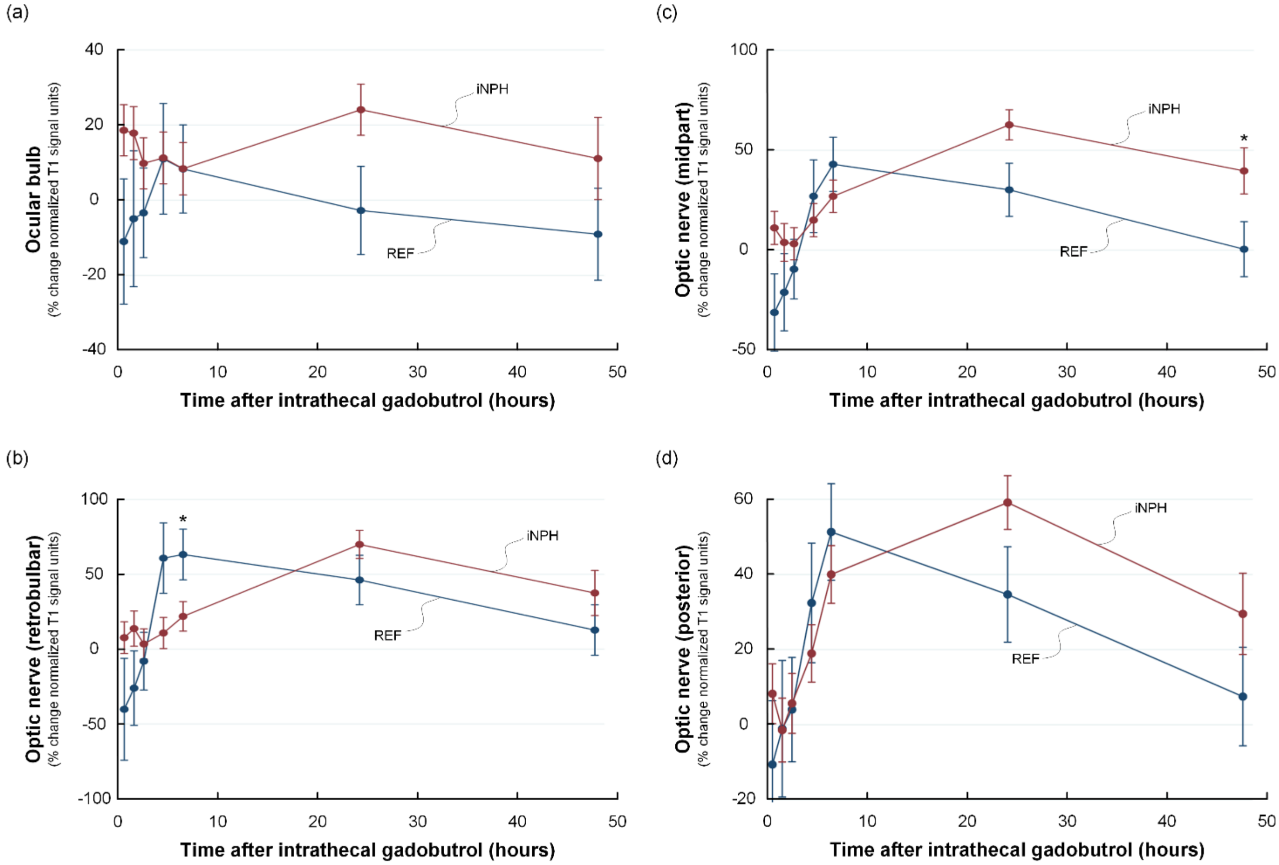

Suppl. Fig. 2. Percentage change in tracer enrichment within intraorbital segment after adjusting for differences in age between groups. The results were analyzed with multivariate analysis using age as a main effect (covariate) in the model. Given that age has a main effect on the results, the model adjusts for age-differences between the REF and iNPH groups. From the multivariate analysis model, trend plots were adjusted to age 65 years in both groups for the locations (a) vitreous body, (b) retrobular part of the optic nerve, (c) mid part of the optic nerve, and (d) posterior part of the optic nerve. The overall P-value for age was non-significant for all locations. Data presented as mean with standard error. *p<0.05, **P<0.01; multivariate analysis.

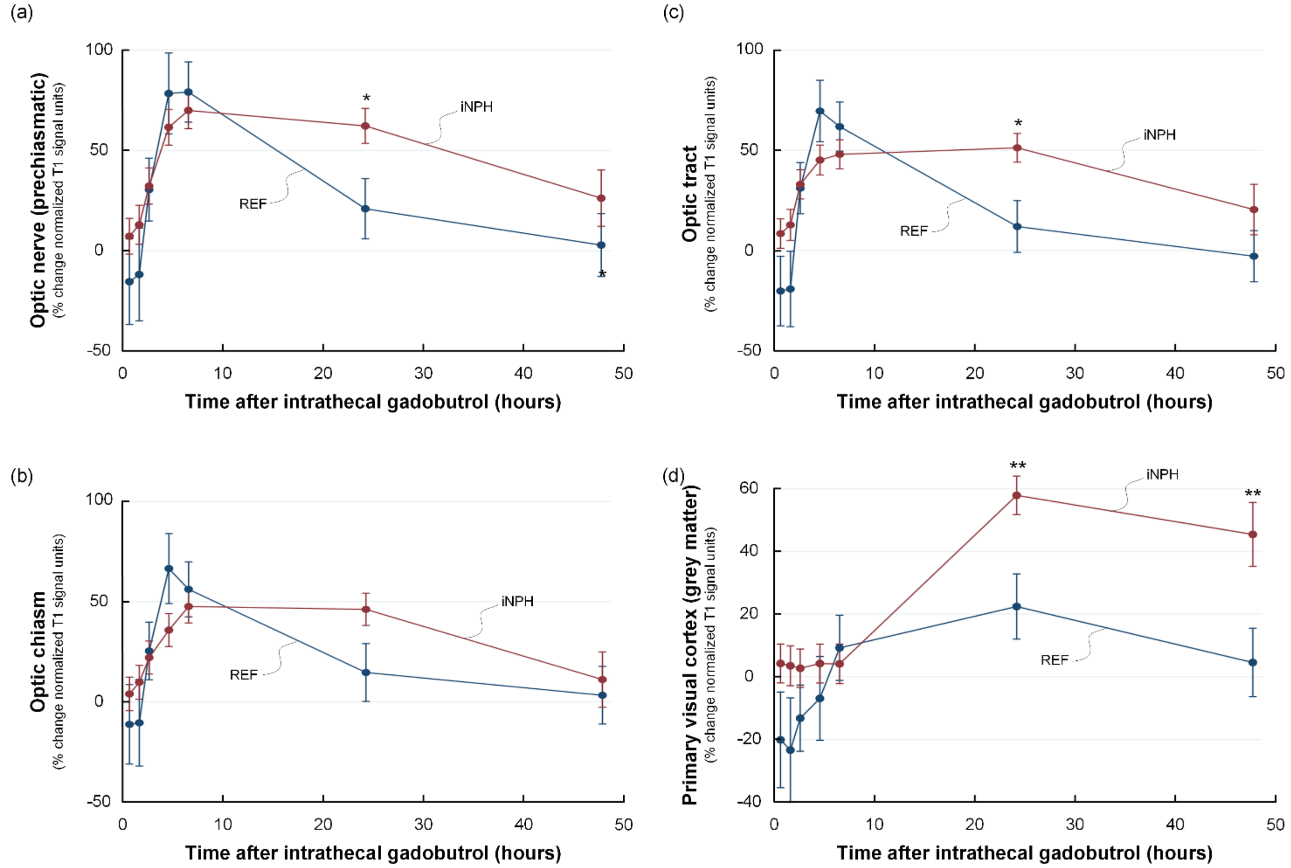

Suppl. Fig. 3. Percentage change in tracer enrichment within intracranial segment after adjusting for differences in age between groups. The results were analyzed with multivariate analysis using age as a main effect (covariate) in the model. Given that age has a main effect on the results, the model adjusts for age-differences between the REF and iNPH groups. From the multivariate analysis model, trend plots were adjusted to age 65 years in both groups for the locations (a) prechiasmatic part of the optic nerve, (b) the optic chiasm, (c) the optic tract, and (d) the primary visual cortex. The overall P-value for age was non-significant for all locations. Data presented as mean with standard error. *p<0.05, **P<0.01; multivariate analysis.
